# Network analysis of comorbid aggressive behavior and testosterone among bipolar disorder patients: a cross-sectional study

**DOI:** 10.1038/s41398-024-02957-1

**Published:** 2024-05-29

**Authors:** Yi Liu, Hong Cai, Tian Han, Yi-Fan Wang, Juan Li, Xiao-meng Xie, Xiao Ji

**Affiliations:** 1 Beijing Key Laboratory of Mental Disorders, National Clinical Research Center for Mental Disorders & National Center for Mental Disorders, Beijing Anding Hospital, & the Advanced Innovation Center for Human Brain Protection, Capital Medical University, Beijing, China; 2 Unit of Psychology Medicine and Behavior Medical, School of Public Health, Guangxi Medical University, Nanning, Guangxi China

**Keywords:** Bipolar disorder, Prognostic markers

## Abstract

Testosterone has complex effects on psychological traits and behavior; it is associated with social dominance and competition and is a potential human sex pheromone. This study aimed to investigate the associations between testosterone levels, aggressive behavior, and manic symptoms using a network analysis among bipolar disorder (BD) patients in psychiatric emergency departments (PED). Data from January 2021 and March 2022 BD patients in PED were analyzed. Manic symptoms were assessed using the Young Mania Rating Scale (YMRS). Aggression was assessed with subscale of the PANSS scale (PANSS-AG). The undirected network structures of testosterone levels, aggressive behavior, and manic symptoms were estimated, and centrality and bridge centrality indices were examined. Network stability was examined using the case-dropping procedure. The Network Comparison Test (NCT) was conducted to evaluate whether network characteristics differed by gender. We recruited a total of 898 BD patients, with the mean YMRS score as 13.30 ± 9.58. The prevalence of level II aggression was 35.6% (95%CI = 32.5%–38.7%), level III aggression was 29.5% (95%CI = 26.3%–32.6%), and level VI aggression was 7.0% (95%CI = 5.4%–8.8%). The male participants had a mean testosterone level of 391.71 (Standard Deviation (SD):223.39) compared to 36.90 (SD:30.50) for female participants in the whole sample. Through network analysis, “Increased motor activity-energy” emerged as the central symptom, with the highest centrality expected influence, followed by “Emotional Instability” and “Disruptive/aggression behavior”. Notably, “Emotional Instability” appeared to be the bridge symptom linking manic symptoms to aggressive behavior. Within the flow network model, “Speech rate and amount” exhibited the strongest positive correlation with testosterone levels, followed closely by “Disruptive/aggression behavior”. The constructed network model demonstrated robust stability, with gender showing no significant impact on the structure. In this study, “Increased motor activity-energy” stood out as the most influential symptom, and “Speech rate and amount” acted as the main bridge symptom linking testosterone levels, aggressive behavior, and manic symptoms. Targeting the central and bridge symptoms may improve the outcomes of aggression interventions implemented among BD patients in psychiatric emergency care.

## Introduction

Bipolar disorder (BD) is a potentially lifelong condition characterized by extreme changes in mood with high incidences of hyperactivity, irritability, grandiosity, poor judgment, and other symptoms, all of which are highly likely to lead to aggressive behavior [1]. Impulsive aggression is common amongst BD patients. Notably, aggressive behavior in BD patients show a direct prognostic value, which links to suicidal behaviors, more frequent hospitalizations, higher severity of mania symptoms, mixed symptoms, and comorbid borderline personality disorder [2]. A characteristic of BD emerges as hindrance towards an individual’s ability to perceive risk and protect themselves, which make them vulnerable to physical assault [3]. Given the risk of hurting others and self-harm, patients with BD have profound effects on society and family numbers.

The etiology of BD is unknown; however, factors such as genetics, biological traits, and environment have been proposed for consideration in the pathogenesis of BD [4]. For patients with BD who are experiencing disease episode, emergency departments or psychiatric emergency department (PED) are often their preferred choice for medical treatment. The highest prevalence of aggressive behavior occur in acute care settings (12.5–61.8%) [5, 6]. Psychiatric emergency department is suitable place to study the relationship for testosterone levels, aggressive behavior, and manic symptoms among patients with BD.

Several lines of evidence indicates that testosterone may be involved in the pathophysiology of aggressive behavior [7, 8]. Testosterone, an androgenic steroid hormone that is regulated by the hypothalamus-pituitary-gonadal (HPG) axis, has traditionally been associated with the manifestation of aggression, partly due to its close relationship with dominance behavior and competitive tendencies [9]. A study reported that administering exogenous testosterone can rapidly increase aggressive behavior in adult males [10]. Testosterone maintains have a complex relationship with BD; it can impact emotions, with elevated levels of testosterone associated with increasing incidences of depressive and hypomania symptoms [11]. Some gender differences have been observed with respect to the clinical course in patients with BD [12]. This evidence suggests a relationship between testosterone levels and aggressive behavior need to be discuss.

Hence, we need to establish a interaction assessment model, which is helpful for understanding the pathogenesis of aggressive behavior in patients with BD. Clinicians could then establish suitable biomarkers for the purposes of screening, risk appraisal, and subsequent therapeutic interventions [13]. In recent years, network analysis has experienced an expanding application in the field of psychology and psychiatry [14]. Network analysis is an innovative and analytical method that elucidates a biological system by anticipating the inter-relationships among multiple syndromes [15]. To further probe the inter-relationship between testosterone and aggressive behavior in BD patients, we incorporated network analysis into this investigation and identify symptoms as robust central symptoms, bridge symptoms and short paths between testosterone and aggrresive behavior within such populations in psychiatric emergency care. We hypothesize that high levels of testosterone may represent a key link point in neurobiological activity, which might contribute to aggression by exacerbating emotional instability.

## Methods

### Patients and study sites

A cross-sectional survey was conducted between January 2021 and March 2022 in Beijing Anding Hospital’s psychiatric emergency department, which is the only 24-h psychiatric hospital emergency service in the Beijing municipality and neighboring provinces. During the study, all patients who had received psychiatric emergency service were recruited consecutively for the survey. Eligible participants had to meet the following criteria: (1) receiving emergency maintenance treatment for a major psychiatric disorder; (2) provided written informed consent; (3) clinical diagnosis of a manic episode (F30–F39 in the ICD-10) (i.e., the Young Mania Rating Scale (YMRS) total score of ≥20 (Ouyang et al. [41]). Ethical approval was obtained from the Ethics Committee of Beijing Anding Hospital.

The basic socio-demographic and clinical data (e.g., age, onset age, gender, education level, marital status, employment status, illness duration, family history of psychiatric disorders) were collected using a form designed for this study through a review of medical records and confirmation from a clinical interview.

### Blood collection and assays of testosterone

The routine blood collections were performed for clinical evaluations during psychiatric emergency visits. Serum samples were collected from all patients between 7:30 AM and 8:30 AM. Controlling for age and gender, the levels of 8 (7.30–8.30) AM serum were assayed for Testosterone (μg/dl) using chemiluminescence. The laboratory personnel were blind to all clinical information. Samples and data were processed following standard operating procedures with the appropriate approval of the Ethics and Scientific Committee of Capital Medical University, and all subjects provided written informed consent.

### Measurement

In this study, aggressive behavior was assessed through interviews conducted by a trained attending psychiatrist within 12 h after admission. The aggressiveness of the subjects was graded using the standardized scale delineated by the local health authority and extensively employed in Chinese clinical practice. The severity of aggression is assessed in three domains: verbal aggression, aggression towards property, and physical aggression towards individuals. Verbal aggression, in this context, pertains to yelling or screaming, exhibiting hostile or offensive gestures, or uttering profanities. The standardized scale determines the intensity of each possible form of aggression on a scale of I to VI, with a level above II considered as aggression. We also used the aggression subscale of the PANSS scale (PANSS-AG), which includes supplementary components for the risk aggression profile (such as anger, difficulty in delaying gratification, and emotional instability) [16]. The PANSS had been validated in the Chinese population and demonstrates satisfactory psychometric properties (Cronbach’s alpha = 0.84) [17].

The Youth Mania Rating Scale (YMRS) [18] is an 11-item clinical rating scale used to assess the severity of manic symptoms. Seven of the eleven individual YMRS items were scored on a 0–4 scale: appearance, insight, language-thought disorder, increased motor activity-energy, elevated mood, sleep, and sexual interest, while the remaining four items were scored on a 0–8 scale: disruptive/aggressive behavior, content of morbid thinking, irritability, and speech–rate and amount. The YMRS total score, with a range of 0–60, is a summation of each of the eleven individual scores, with higher total scores signifying a more severe manifestation of mania.

### Statistical analyses

Data were analyzed with the IBM Statistical Package for Social Science (SPSS) software version 24.0 and R software version 4.2.3. Normality of the data was assessed using the Kolmogorov–Smirnov test. Using the R software [19], a network model of aggression severity and testosterone level was built. To examine the edges of the network, we computed the polychoric correlations between all items and estimated the Graphical Gaussian Model (GGM) using the graphic least absolute shrinkage and selection operator (LASSO), and the Extended Bayesian Information Criterion (EBIC) using the R package graph [20].

The importance of each node in the network was examined by estimating centrality indices of the network structure with the R package “graph” [21]. Specifically, the centrality index of expected influence (EI) was computed for each node in the network (i.e., the sum of the weights of the connections, in absolute value), because EI is the most stable and interpretable centrality index [20]. The thickness of the edge represents the strength of the association. Additionally, previous studies [22] on comorbid psychiatric syndromes found that “Testosterone” was commonly reported to link different symptom communities as a key node. Therefore, the node-specific predictive betweenness of “Testosterone” (i.e., how often a node lies on the pathways between two other nodes, always with the “Testosterone” node as either of them across 1000 nonparametric bootstrap iterations) was estimated [23]. To identify particular symptoms that were directly associated with “Testosterone”, the “flow” function in the R package ‘qgraph’ was used [21].

Following previous studies [24, 25], the differences in network characteristics between male and female participants were compared using the R “NetworkComparisonTest” package (Version 2.2.1) [26] with 1000 permutations. The difference in network structure (e.g., distributions of edge weights), global strength (e.g., total absolute connectivity among the symptoms), and each specific edge between subsamples (i.e., females vs. males) were also examined.

## Results

A total of 915 patients were invited to participate in this study; 898 patients met the study criteria and were included in analyses, in which the response rate was 98.1%, the rest of them were not included in the analysis due to incomplete information. The prevalence of level II aggression was 35.6% (95%CI = 32.5%–38.7%), level III aggression was 29.5% (95%CI = 26.3%–32.6%), and level VI was 7.0% (95%CI = 5.4%–8.8%). The male participants had a mean testosterone level of 391.71 ± 223.39 ng/dl (reference range 260–1590 ng/dl for ♂) compared to 36.90 ± 30.50 ng/dl for female participants (reference range 15–80 ng/dl for ♀). The demographic and clinical characteristics of the study population are summarized in Table 1.

**Table 1:** The social-demographic and clinical characteristics.

Variables	Total	Male	Female	Univariate analyses		
	N (%)	N (%)	N (%)	*χ*^2^	df	*P*
Employed	471 (52.4)	274 (51.5)	197 (53.8)	0.47	1	0.49
Medical insurance	647 (72.0)	384 (72.2)	263 (71.9)	0.01	1	0.92
Unmarried	398 (45.9)	244 (42.1)	154 (44.3)	18.92	1	0.09
Family history	238 (26.5)	144 (27.1)	94 (25.7)	7.36	1	0.06
	**Mean (SD)**	**Mean (SD)**	**Mean (SD)**	***t***	**df**	***P***
Age (years)	39.4 (16.1)	39.4 (17.3)	39.5 (16.2)	1.18	896	0.24
Education background (years)	13.1 (3.8)	13.0 (3.5)	13.2 (3.7)	-0.62	896	0.54
First episode age (years)	31.8 (14.5)	31.9 (15.9)	31.5 (14.2)	1.19	896	0.23
	**Median(IQR)**	**Median(IQR)**	**Median(IQR)**	***Z***	**df**	***P***
BMI	23.1 (6.07)	22.4 (5.95)	23.01 (5.67)	−0.10	--	0.92
Illness duration (months)	14.0 (6.0)	15.0 (7.0)	12.0 (4.0)	−1.36	--	0.17
YMRS total	13.0 (19.0)	15.0 (20.0)	11.0 (19.0)	−0.52	--	0.60

### Network structure

**Fig. 1:**
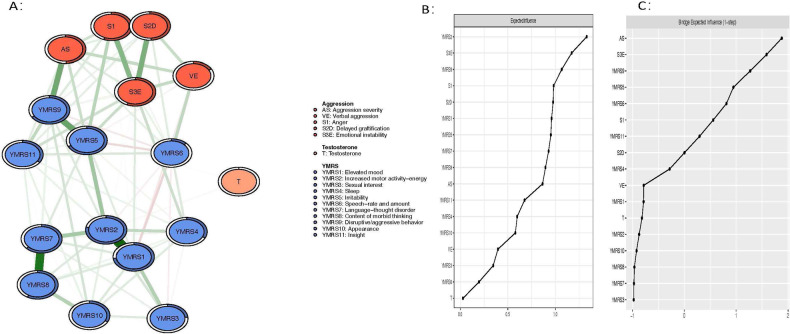
Network analysis of comorbid aggression severity, Manic symptoms, and Testosterone among patients with mood disorders. AS aggression severity, FE facial expression, SI anger, S2D delyed graftification, S3E Emotional instability, T testosterone, YMRS1 high mood, YMRS2 increased behavioral activity, YMRS3 sexual interest, YMRS4 sleep, YMRS5 ittitability, YMRS6 language speed and quantity, YMRS7 language thinking disturbed, YMRS8 patients thinking, YMRS9 attack and sabotage, YMRS10 appearance, YMRS11 self-control.

Figure 1 illustrates the network structure representing the comorbid severity of aggression, manic symptoms, and testosterone levels in participants diagnosed with BD. The network model reveals that the strongest positive association among manic symptoms is between YMRS1 (“Elevated mood”) and YMRS2 (“Increased motor activity-energy”), followed by YMRS7 (“Language-thought disorder”) and YMRS8 (“Content of morbid thinking”), as well as YMRS5 (“Irritability”) and YMRS9 (“Disruptive/aggression behavior”).

**Fig. 2:**
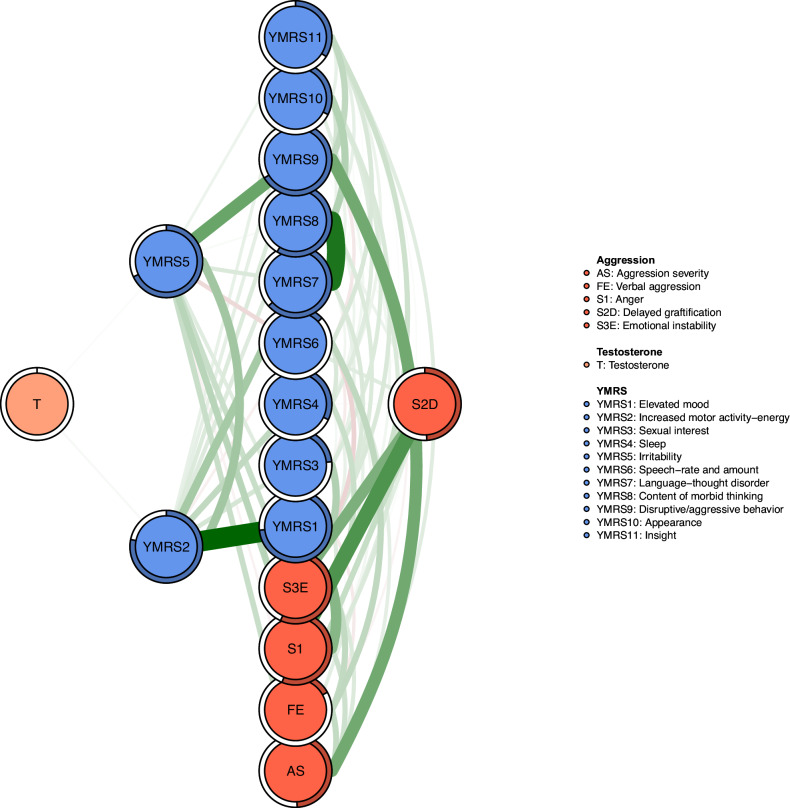
Flow network of Testosterone.

Regarding EI centrality, the node YMRS2 (“Increased motor activity-energy”) exhibits the highest value, followed by S3E (“Emotional instability”) and YMRS9 (“Disruptive/aggression behavior”) within the network (Fig. 1). In terms of bridge EI, S3E (“Emotional instability”) emerges as the most critical bridge symptom connecting aggression and manic symptoms, succeeded by YMRS9 (“Disruptive/aggression behavior”) and S2D (“Delaying gratification”) (Fig. 1). Furthermore, the flow network model demonstrates that YMRS6 (“Speech–rate and amount”) exhibits the strongest positive correlation with testosterone, followed by YMRS1 (“Elevated mood”) (Fig. 2). In addition, we found that YMRS6 (“Speech–rate and amount”) has the strongest positive association with testosterone in the flow network model, followed by the YMRS1 (“Elevated mood”) (Fig. 2).

**Fig. 3:**
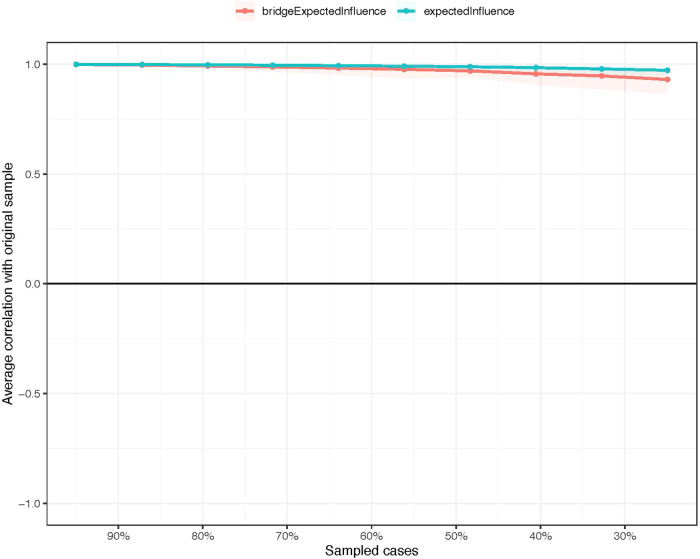
The stability of centrality and bridge centrality indices using case-dropping bootstrap.

Regarding network stability, the EI centrality demonstrates an exceptional level of stability (i.e., CS-coefficient = 0.75 (95% CI: 0.675-1)), indicating that the network structure would not change significantly even if 75% of the sample was removed (Fig. 3). The bootstrap difference test revealed that most comparisons between edge weights were statistically significant.

### Node-specific predictive betweenness measure

**Fig. 4:**
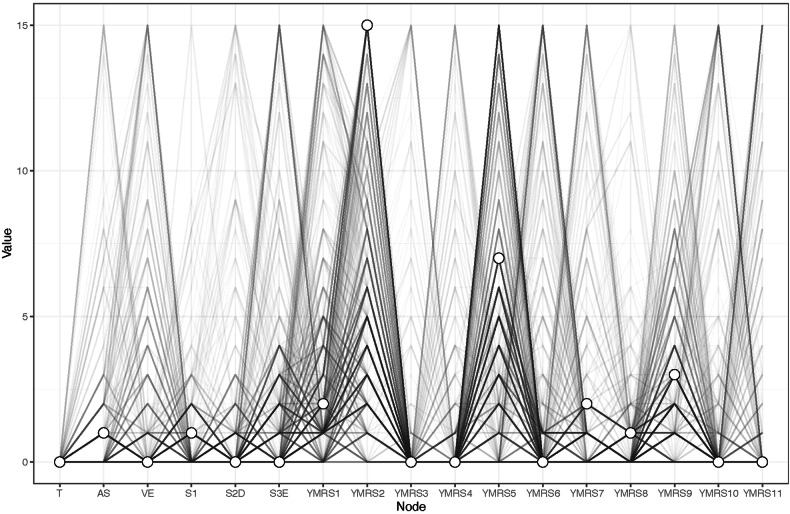
Node-specific predictive betweenness.

Researchers have found that “Testosterone” plays an important role in aggressive behavior in previous studies [22]. Figure 4 shows the node-specific predictive betweenness values for each node in the network. The white dots represent the node-specific predictive betweenness in the study sample, while the black lines represent the variability of the measure across 1000 nonparametric bootstrap iterations. YMRS6 (“Speech–rate and amount”) has the highest node-specific predictive betweenness score, followed by YMRS5 (“Irritability”). This finding suggests that “Speech–rate and amount” and “Irritability” may be the main bridge symptoms between testosterone levels, manic symptoms, and aggression (Fig. 4).

### Network comparison tests by gender

The comparison of the network model by gender did not reveal significant differences in network global strength (network strength: 11.37 in female participants; 11.40 male participants; M = 0.214, P = 0.571) and edge weights (S = 0.026, P = 0.969, Supplementary Figs. S1–S4).

## Discussion

The study is the first network analysis of the association between testosterone levels and aggressive behavior among patients with BD in China PED. Within the assessed network model, “Increased motor activity-energy”, “Emotional instability” and “Disruptive/aggressive behavior” stood out as the most influential symptoms. Notably, “Speech rate and amount” acted as the main bridge symptom linking testosterone levels, manic symptoms, and aggression, followed by “Elevated Mood” and “Difficulty in delaying gratification”.

Our network analysis highlighted “Increased motor activity-energy” and “Emotional instability” as central symptoms in patients arriving at the emergency room due to aggressive tendencies. Patients with BD often displays alcohol or substance abuse, which can increase behavioral activity, leadings to an increased prevalence of aggressive behavior possibly up to 12% [27]. Consistent with previous findings, aggression was associated with more severe manic symptoms, as measured by the YMRS in acute manic episodes [28]. People in the manic phase are often highly motivated to engage in goal-oriented activities. However, due to lack of insight, patients may not realize their behavior is abnormal and prone to harmful consequences. Patients become extremely aggressive and irritable, and are more likely to harm themselves or others and destroy property through verbal or physical aggression [29]. This observation also shed light on the identification of “Difficulty in delayed gratification” as another bridge symptom. Previous functional magnetic resonance imaging (fMRI) studies have observed responses in the amygdala, periaqueductal gray matter, and the prefrontal lobe when BD patients were frustrated by the denial of a reward. This neural response can potentially stimulate aggressive impulses, transforming unfulfilled desires into the urge to attack [30].

“Speech–rate and amount” acted as the main bridge symptoms linking testosterone levels, manic symptoms, and aggression, succeeded by “Elevated Mood”. “Speech–rate and amount” and “Elevated mood” are typical symptoms of manic episodes, and previous imaging studies have shown abnormalities in neuronal coupling in the sensory-motor subcortical-cortical circuit in patients with highly excited brains, compared to healthy controls [31]. Testosterone may act through receptors located in key regulatory regions to increase the connectivity between subcortical regions of the brain while weakening connections between the cortex and subcortical regions [32], causing the patient in highly excited state. Abnormal regulation of the HPG axis is critical for homeostatic regulation of synthesis and secretion of testosterone and the most potent androgen dihydrotestosterone (DHT) by the testis [33]. Dysregulation of the HPG axis may cause depressive symptoms, which are associated with high cortisol inhibition [34]. Moreover, compared to bodybuilders who did not take exogenous testosterone, there was a higher rate of hypomanic episodes among those who did [35]. The release of testosterone will increase the motivation of BD patients to seek dominance and take impulsive risks, which are often poorly thought-out and prone to adverse consequences [36]. High levels of testosterone have been found to reduce activity and functional connectivity in the prefrontal cortex, altering the function of prefrontal-mediated emotion regulation, thereby impairing the ability to control aggressive impulses [37].

Regarding the gender for the network model of comorbid aggression severity, manic symptoms, and testosterone levels among patients with BD, there were none significant differences shown in this study. The existing evidence suggests that testosterone concentrations in early life influence the development of some human behaviors, with certain gender differences [38]. One study from community recruiters suggested that adolescent males with higher plasma testosterone levels were more irritable and likely to overreact to provocation and threats [39]. Previous studies on depressive episode status in patients with BD have found that male patients have significantly lower testosterone levels, while female patients have significantly higher levels [40].

The strengths of this study include a substantial sample size, the utilization of standardized assessment instruments, and the employment of network analysis techniques. These techniques were tailored to investigate the structure of the model assessing testosterone levels and aggressive behavior in patients with BD. Such insights may pinpoint potential targets for treatment and prevention measures, particularly for patients with BD in psychiatric emergencies exhibiting these problems. Nonetheless, several methodological limitations in our study warrant attention. First, the cross-sectional design precludes establishing causal relationships between variables, and there was an absence of a control/naive group. Future longitudinal research is essential to elucidate the time-bound causal links between testosterone levels and aggressive behavior in patients with BD. Secondly, though routine blood collections were performed for clinical evaluations during psychiatric emergency visits, additional measurements, such as thyroxine, should be added to the study analysis. Subsequently, we hope to carry out relevant radiological research to further explore the specific relationship between testosterone levels and aggressive behavior in BD patients. Lastly, our sample was consecutively drawn from one major psychiatric emergency department (PED) in China, which may limit the generalizability of our findings in other psychiatric contexts.

To summarize, within the aggression-bipolar disorders network model, as well as the association with testosterone levels, “Increased motor activity-energy,” “Emotional instability,” and “Disruptive/aggressive behavior” were the most pivotal symptoms among BD patients in PED. Our study showed no significant gender differences in testosterone levels observed in BD patients during the manic episode phase. These specific symptoms, which are potential intervention targets, should be at the forefront of physicians’ evaluation protocols.

### Supplementary information

supplementary materials

## Data Availability

The data of the investigation will be made publicly available if necessary.
