# Supplementary material for: Network analysis of comorbid aggressive behavior and testosterone among bipolar disorder patients: a cross-sectional study
Source: Transl Psychiatry. 2024 May 29;14:224. doi: 10.1038/s41398-024-02957-1 (PMC11137147; doi:10.1038/s41398-024-02957-1)

**Supplementary materials**

Figure S1. Estimated network model for aggression severity, Manic symptoms and Testosterone among females and males

Figure S2. Comparison of network flow of Testosterone between females and males

Figure S3. Comparison of Node-specific predictive betweenness in females and males

Figure S4. Comparison of network properties between females and males

Figure S1. Estimated network model for aggression severity, Manic symptoms and Testosterone among females and males


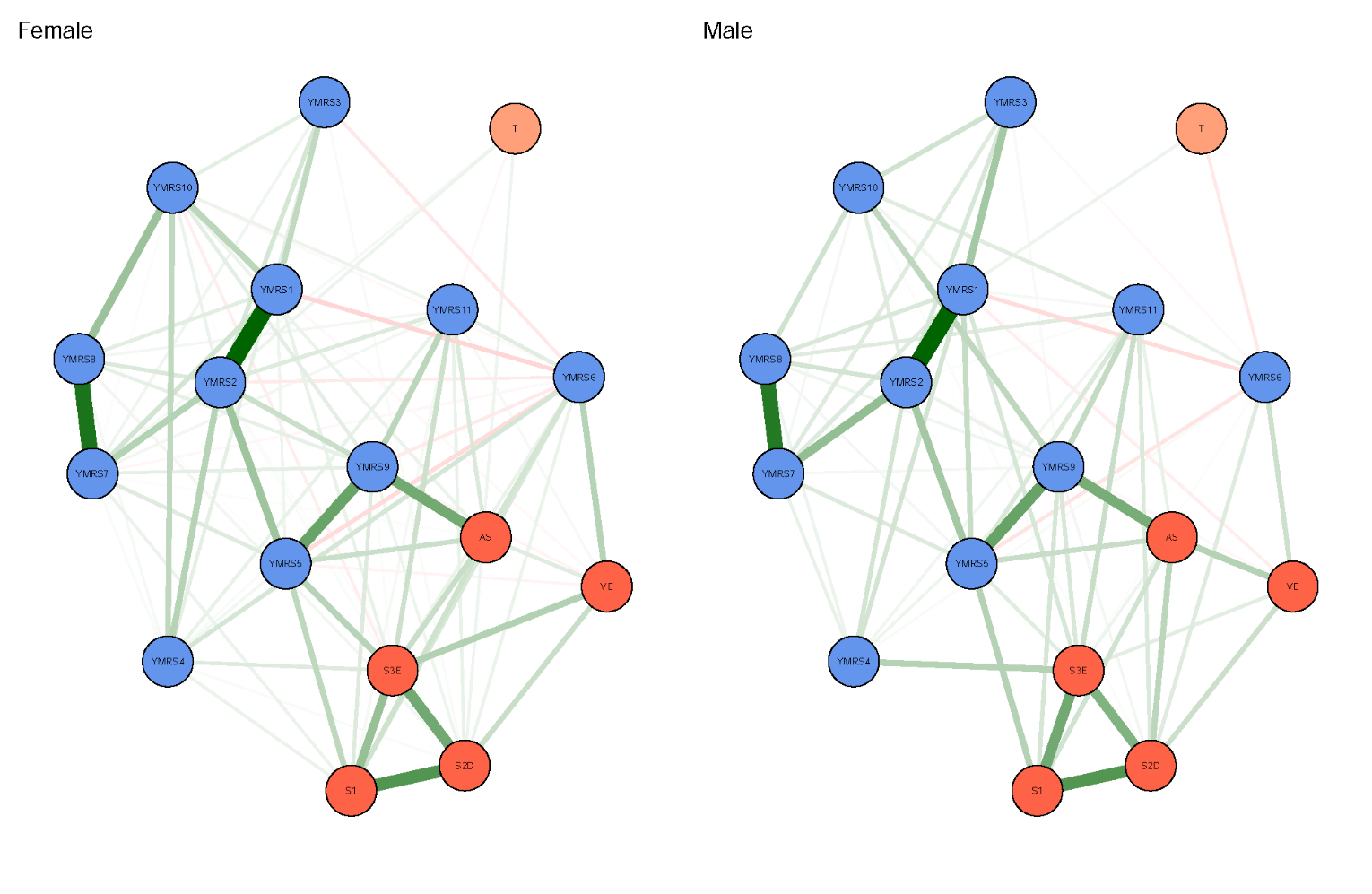


Figure S2. Comparison of network flow of Testosterone between females and males


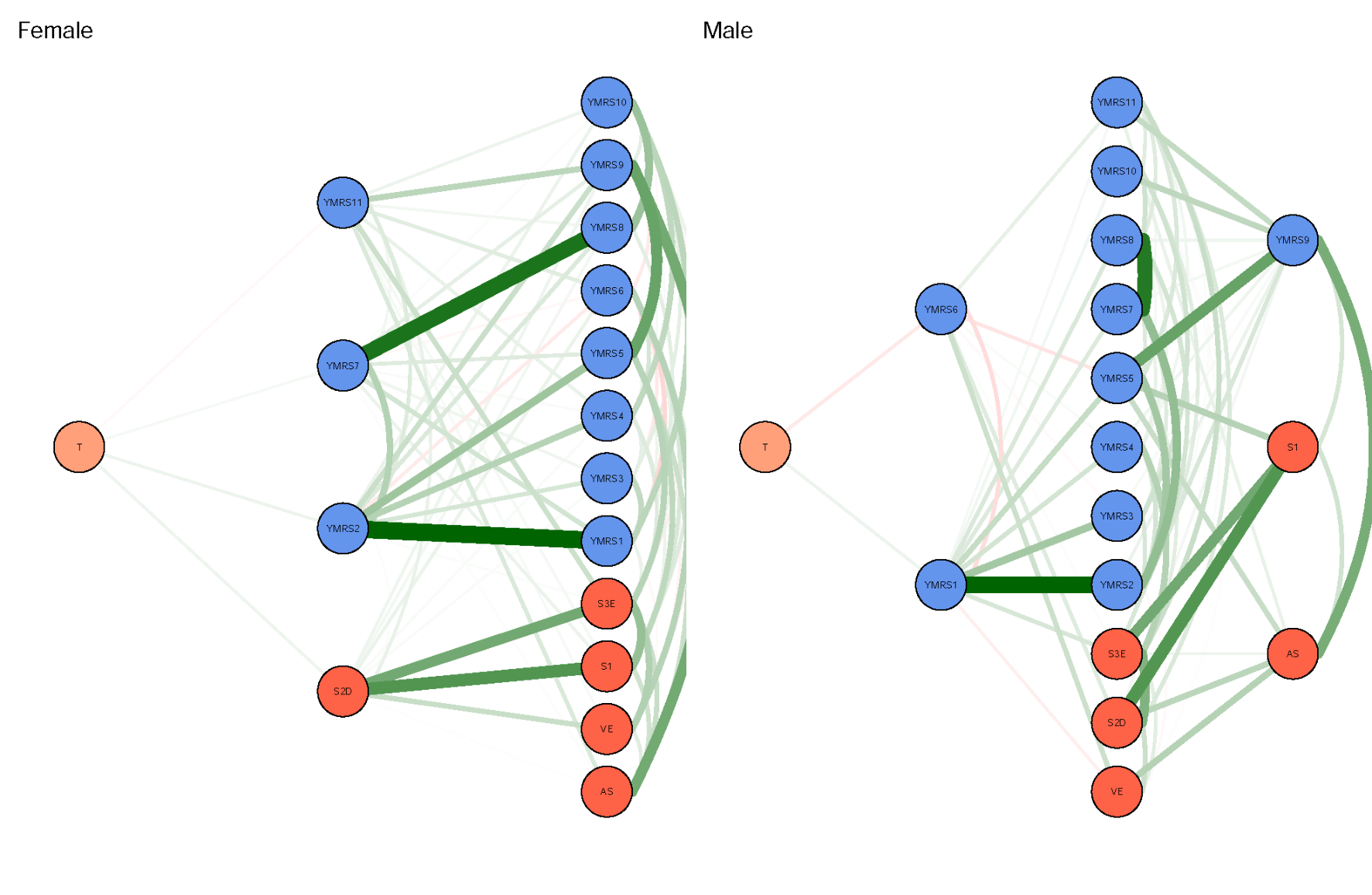


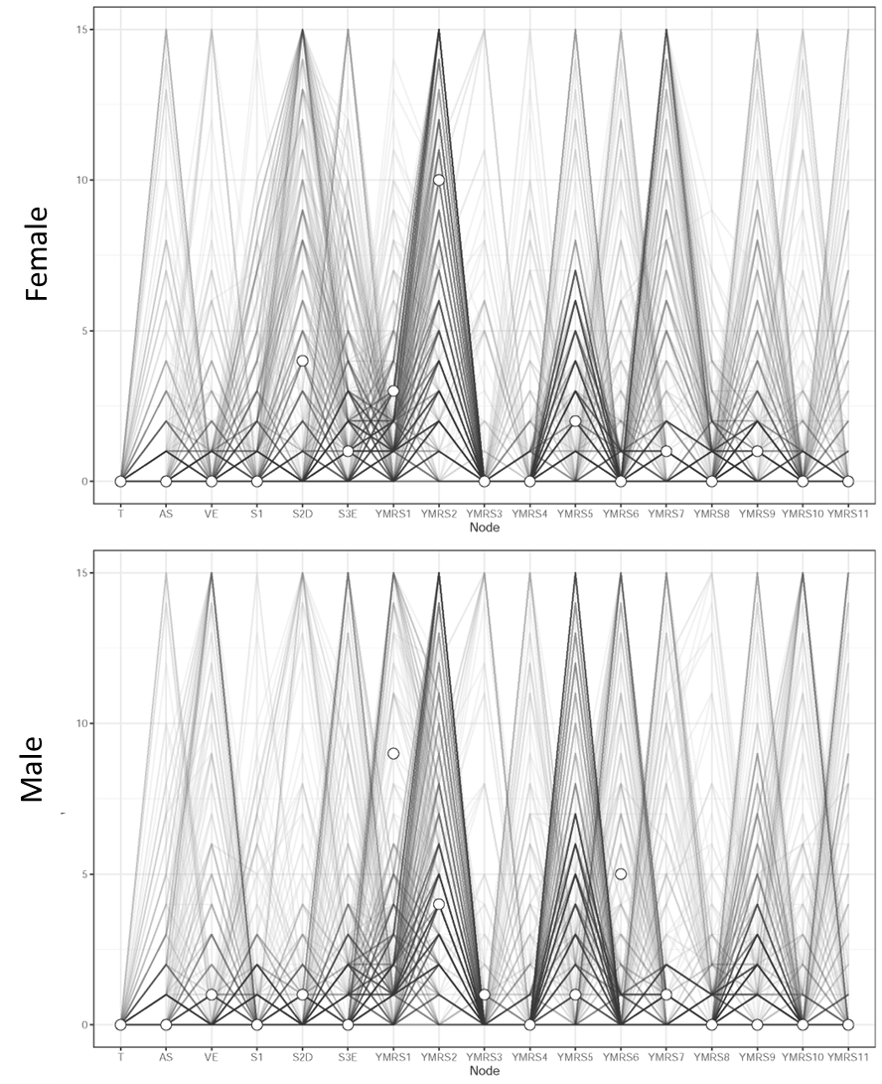
Figure S3. Comparison of Node-specific predictive betweenness in females and males

Figure S4. Comparison of network properties between females and males


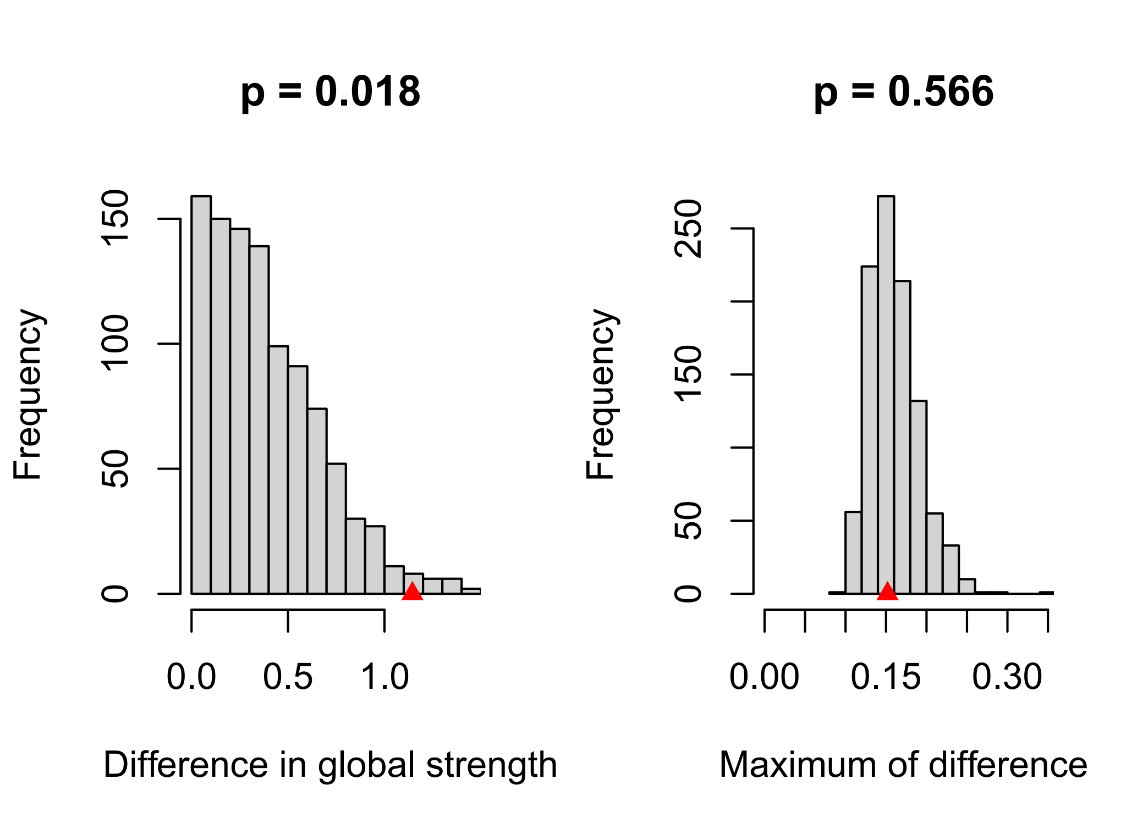

Supplement: Supplementary file 1 — supplementary materials [file 41398_2024_2957_MOESM1_ESM.docx]
